# PROMOTING ELDER JUSTICE IN THE VETERANS HEALTH ADMINISTRATION VIA INTER- AND EXTRADEPARTMENTAL COLLABORATIONS

**DOI:** 10.1093/geroni/igad104.0422

**Published:** 2023-12-21

**Authors:** Lena Makaroun, Kathrine Smith, Lisa Boris, Jaime Halaszynski

**Affiliations:** University of Pittsburgh School of Medicine, Division of Geriatric Medicine, Pittsburgh, Pennsylvania, United States; VA Fargo Health Care System, Fargo, North Dakota, United States; VA Northeast Ohio Healthcare System, Ravenna, Ohio, United States; VA Butler Health Care System, Butler, Pennsylvania, United States

## Abstract

The Veterans Health Administration (VHA), the largest integrated health system in the country, provides comprehensive, high-quality care to nearly 19 million Veterans, over half of whom are age ≥60. Older Veterans receiving care in VHA have a high prevalence of most known elder abuse (EA) risk factors, many stemming from their military service. The VHA is a leader in advancing knowledge and care for complex geriatric syndromes, and now leaders in both VHA research and social work are spearheading interdisciplinary collaborations to do the same for EA. In this symposium, we present practical experiences, empirical data and lessons learned from four VHA led collaborations, including: 1) development of the VHA National Social Work Elder Abuse Tiger Team that has worked with VHA researchers to develop and lead a national strategy around EA response; 2) a partnership with the Veterans Benefits Administration to conduct novel research on financial exploitation among Veterans; 3) an education and training collaboration with the National Adult Protective Services Association to demystify the processes and procedures of each organization that has thus far led to 7 presentations to >2,500 attendees; and 4) a relationship with the National Collaboratory to Address Elder Mistreatment to adapt and pilot an EA screening tool for use in VHA emergency departments. Highlighting how the VHA has built bridges both within and outside its organization and the impact this has had on advancing EA research and response will generate ideas for other healthcare systems and those interested in future collaborations with VHA.

